# Experimental observation of nonadiabatic bifurcation dynamics at resonances in the continuum†
†Electronic supplementary information (ESI) available. See DOI: 10.1039/c8sc04859b


**DOI:** 10.1039/c8sc04859b

**Published:** 2019-01-04

**Authors:** Jean Sun Lim, Hyun Sik You, So-Yeon Kim, Sang Kyu Kim

**Affiliations:** a Department of Chemistry , KAIST , Daejeon 34141 , Republic of Korea . Email: sangkyukim@kaist.ac.kr

## Abstract

The surface crossing of bound and unbound electronic states in multidimensional space often gives rise to resonances in the continuum. This situation happens in the πσ*-mediated photodissociation reaction of 2-fluorothioanisole; optically-bright bound S_1_ (ππ*) vibrational states of 2-fluorothioanisole are strongly coupled to the optically-dark S_2_ (πσ*) state, which is repulsive along the S–CH_3_ elongation coordinate. It is revealed here that the reactive flux prepared at such resonances in the continuum bifurcates into two distinct reaction pathways with totally different dynamics in terms of energy disposal and nonadiabatic transition probability. This indicates that the reactive flux in the Franck–Condon region may either undergo nonadiabatic transition funneling through the conical intersection from the upper adiabat, or follow a low-lying adiabatic path, along which multiple dynamic saddle points may be located. Since 2-fluorothioanisole adopts a nonplanar geometry in the S_1_ minimum energy, the quasi-degenerate S_1_/S_2_ crossing seam in the nonplanar geometry, which lies well below the planar S_1_/S_2_ conical intersection, is likely responsible for the efficient vibronic coupling, especially in the low S_1_ internal energy region. As the excitation energy increases, bound-to-continuum coupling is facilitated with the aid of intramolecular vibrational redistribution, along many degrees of freedom spanning the large structural volume. This leads to the rapid domination of the continuum character of the reactive flux. This work reports direct and robust experimental observations of the nonadiabatic bifurcation dynamics of the reactive flux occurring at resonances in the continuum of polyatomic molecules.

## Abstract 2

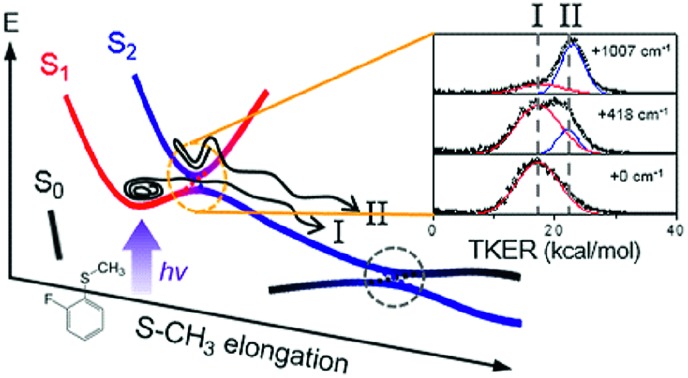
In the strong vibronic state mixing regime, both Herzberg type-I and type-II predissociations coexist and proceed in a competitive way.

## Introduction

Resonances in the continuum are frequently met in the excited-state dynamics of photodissociation, ionization, or photodetachment processes.^1^–^4^ The coupling of discrete and continuum states gives rise to quantum-mechanical interferences when coherently excited states interfere with each other along distinct pathways that lead to an identical final destination. Though a Fano-type resonance has often been reported in atomic systems, it has rarely been observed for polyatomic molecules, for only a few examples including H_3_,^5^ FNO^6^–^9^ or CH_2_N_2_.^10^ In some sense, this could be natural since the reaction dynamics in polyatomic systems occur on complicated multidimensional potential energy surfaces. This can hamper the observation of quantum interferences, since so many degrees of freedom are involved in the bound-continuum coupling, and the coherence volume pertaining to a chemical reaction becomes extremely small compared to the entire phase space experienced in resonances in the continuum. But while quantum interferences have rarely been observed, bound-to-continuum coupling in polyatomic system is ubiquitous in a number of chemical and biological units, and it is believed that such a coupling is essential to the functionalizing of many important nonradiative processes in nature.^11^,^12^ In recent decades, one of the very intriguing group of polyatomic molecules in this regard, the heteroaromatic molecular systems such as phenols,^13^–^25^ thiophenols,^26^–^36^ or thioanisoles^37^–^46^ have been subject to intensive and extensive studies. Here, chemical bond dissociation occurs *via* coupling of bound (ππ*) to repulsive (πσ*) states, which provides great opportunities to investigate nonadiabatic dynamics, conical intersection, or tunnelling at the atomic level.^11^–^46^ Despite many studies on these systems, however, the detailed mechanism of predissociation on multidimensional potential surfaces in the presence of curve crossings is still beyond our full understanding.

**Fig. 1:**
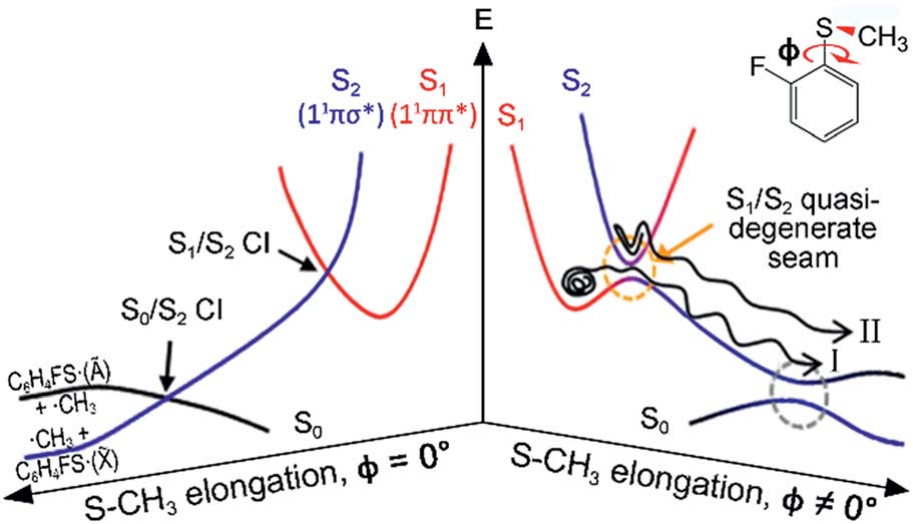
Schematic of potential energy curves for the photodissociation reaction of 2-fluorothioanisole. Two conical intersections (CIs) generated at planar geometries become avoided crossings in the nonplanar structure. The dynamics of 2-fluorothioanisole occurs at *φ* ≠ 0° (the right half) due to the nonplanar excited-state geometry. The bifurcated reaction pathways of channel I and II are depicted (see the text). *φ* is the S–CH_3_ dihedral angle with respect to the plane of benzene moiety.

Even though the S–CH_3_ bond dissociation of thioanisole occurs on complicated multidimensional potential surfaces, one may describe its reaction pathway using a one-dimensional picture. Namely, the optically bright bound S_1_ (^1^ππ*) state is crossed by the near-lying dark S_2_ (^1^πσ*) state, which is repulsive along the S–CH_3_ elongation coordinate, Fig. 1. The S_1_/S_2_ conical intersection seam then acts as a dynamic bottleneck for nonadiabatic transition, through which the reactive flux slides on the repulsive surface to reach the second S_0_/S_2_ conical intersection located in the later stage of the reaction in planar geometry. The reactive flux at the S_0_/S_2_ conical intersection adiabatically correlates to the C_6_H_5_S·(Ã) + ·CH_3_ channel, while the lower-lying C_6_H_5_S·(X[combining tilde]) + ·CH_3_ fragment channel is opened only when nonadiabatic transition occurs through the S_0_/S_2_ conical intersection. For the thioanisole case, our group has reported a surprising resonant feature in the nonadiabatic transition probability observed at a particular S_1_ vibronic mode.^37^ This mode has been identified through a complete spectroscopic analyses to be the 7a mode corresponding to C–S–CH_3_ asymmetric stretching, which is parallel with the gradient difference vector of the S_1_/S_2_ conical intersection. As the dissociation along the S–CH_3_ elongation coordinate is prompt on the repulsive S_2_ surface, the largely enhanced nonadiabatic transition probability persists at the S_0_/S_2_ conical intersection, favouring the C_6_H_5_S·(X[combining tilde]) + ·CH_3_ fragment channel in the asymptotic limit. This thus leads to the resonant-like peak of the X[combining tilde]/Ã branching ratio of the C_6_H_5_S· fragment with the 7a mode excitation of S_1_ thioanisole.

Further experiments on partially-deuterated thioanisole isotope analogues have demonstrated that vibronic modes showing nonadiabatic dynamic resonances provide a unique way of spectroscopically characterizing the S_1_/S_2_ conical intersection seam, by means of S_1_ normal modes within the Franck–Condon optical window, unless intramolecular vibrational redistribution (IVR) intervenes and dilutes mode-specificity.^39^,^40^,^42^ In our recent picosecond time-resolved study, it was revealed that a reactive flux prepared in the proximity of a conical intersection induced by the 7a mode excitation bifurcates into two distinct reaction pathways.^45^ Remarkably, these two reaction pathways are completely different in terms of their reaction rates, energy disposals, and nonadiabatic transition probabilities. According to our interpretation, the faster reaction channel with the larger kinetic energy release and larger X[combining tilde]/Ã ratio originates from the reactive flux residing in the upper adiabat positioned above the S_1_/S_2_ conical intersection, through which the nonadiabatic transition to the repulsive S_2_ surface takes place. Meanwhile, the slower channel with the smaller kinetic energy release and little nonadiabatic transition probability represents the reactive flux seeking the minimum energy path on the low-lying adiabatic potential energy surfaces, leading to the adiabatic product channel of C_6_H_5_S·(Ã) + ·CH_3_ in the asymptotic limit.

Herein, it is revealed that the nonadiabatic bifurcation dynamics found in the proximity of a conical intersection are generally applicable to the reaction dynamics occurring in the strong bound/unbound coupling regime. This provides a robust explanation for the predissociation dynamics taking place at or near resonances in the continuum of polyatomic molecules. In 2-fluorothioanisole, it is experimentally demonstrated that both adiabatic and nonadiabatic pathways coexist for the reactive flux prepared at resonances in the continuum, and they proceed in a competitive way on multidimensional potential energy surfaces. The nonadiabatic bifurcation dynamics strongly depends on vibronic modes and/or total energy, giving essential information about the multidimensional nature of the nonadiabatic coupling dynamics of polyatomic molecules.

## Methods

### Experimental

2-Fluorothioanisole (TCI, >98%) seeded in Ar carrier gas with a backing pressure of 1–1.5 bar was prepared in a supersonic jet through a pulsed nozzle valve (general valve, 0.5 mm diameter). The sample was heated to 55 °C to obtain sufficient vapour pressure. For resonant two-photon ionization (R2PI) spectroscopy, the excitation laser pulse was generated by a frequency-doubled dye laser output (Lambda phisik, Scanmate 2) pumped by a Nd:YAG laser (Surelite II-10). The excitation laser wavelength was scanned from 286 to 270 nm and crossed with the molecular beam, generating parent ions (C_6_H_4_FSCH_3_^+^) when resonant with vibronic transitions. In order to obtain the photofragment excitation (PHOFEX) spectrum, a probe laser pulse was prepared by a tuneable dye laser (Lumonics, HD-500) to ionize the nascent fragment radical ·CH_3_ (*v* = 0) by the (2 + 1) resonance-enhanced multiphoton ionization at 333.45 nm. The pump laser wavelength was scanned while measuring the CH_3_^+^ ion yield. The (1 + 1′) SEVI spectroscopy^47^,^48^ was carried out with two independent tuneable dye lasers, Scanmate 2 and Lumonics. The frequency of the pump laser pulse was fixed to a specific vibronic band of S_1_ state. The second laser pulse was used to generate slow photoelectrons from ionization just above the ionization energy threshold. Ionization laser pulses of at least several different wavelengths were employed to get each SEVI spectrum for a specific S_1_ vibronic band. A cloud of photoelectrons was extracted in the low electric field and mapped onto a microchannel plate (MCP) coupled to a phosphor screen. Images were recorded using a charge-coupled device (CCD) camera (Sony XC-ST50), sent to a PC and processed with the IMACQ acquisition software.^49^ Photoelectron spectra were obtained by angular integration of images reconstructed *via* the BASEX algorithm.^50^ The measured electron kinetic energy was plotted according to the internal energy with respect to the band origin of the cationic ground (D_0_) state. The VMI method^51^ was used to measure the translational energy and angular distributions of the nascent ·CH_3_ fragment. The wavelength of the pump laser was set to an excitation energy in the range of 286–230 nm. After 10–20 ns, the probe laser with 333.45 nm was introduced to detect ·CH_3_ (*v* = 0). The polarization of the both pump and probe lasers was perpendicular to the flight direction of the molecular beam and the ion, but parallel to the plane of the MCP and phosphor screen. The anisotropy parameter (*β*) describes the angular distribution of photofragments: *I*(*θ*) = *σ*/4π[1 + *βP*_2_(cos(*θ*))]. Here, *θ* is the angle of the fragment velocity vector with respect to the polarization vector (**E**) of the pump laser, *P*_2_ is the second-order Legendre polynomial, and *σ* is the absorption cross section.

### Computational

The potential energy curves along *φ* (the dihedral angle between the S–CH_3_ bond and the ring plane) in the S_0_ state were calculated using the MP2 (ref. 52) and B3LYP^53^ methods with a 6-311++G(3df,3pd) basis set. The *φ* value was varied at 10° intervals from 0° to 180° while the rest of the geometrical parameters were being optimized. A natural bond orbital (NBO) analysis^54^ at local minima (*trans* and *gauche* form) in the ground state was performed. The potential energy curves along *φ* for the first three singlet excited states were obtained by calculating vertical excitation energies using the equation-of-motion coupled cluster single and doubles (EOM-CCSD)^55^ level of theory at the respective optimized MP2 geometries. Geometries at the conical intersections were optimized using complete active space self-consistent field (CASSCF)^56^,^57^ with a 6-311++G(3df,3pd) basis set. 12 active electrons and 11 active orbitals consist of three pairs of π/π* orbitals, a nonbonding p orbital of sulfur, σ_S–CH_3__, 
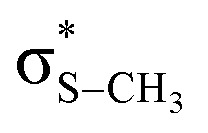
, σ_CS_, and 
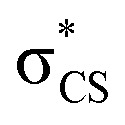
 orbitals. The S_1_ minimum energy was determined by vertical excitation from S_0_ minimum geometry optimized using the same level of theory. The B3LYP and MP2 calculations were performed using Gaussian 09.^58^ The EOM-CCSD and conical intersection optimization calculations were performed using Molpro 2010.1.^59^

## Results and discussion

The reaction dynamics of 2-fluorothioanisole, due to its nonplanar excited-state structure, opens a new aspect of nonadiabatic reaction dynamics by permitting the exploration of the unexplored nuclear configuration space. We carried out spectroscopic characterization of the S_1_ 2-fluorothioanisole to confirm its non-planarity, followed by an investigation of state-selective dynamic outputs. This provides deep insights into the nonadiabatic reaction dynamics occurring in the strong coupling regime of bound and unbound states along the nonplanar nuclear coordinates, which are usually inaccessible in systems undergoing optical transition with little structural changes.

**Fig. 2:**
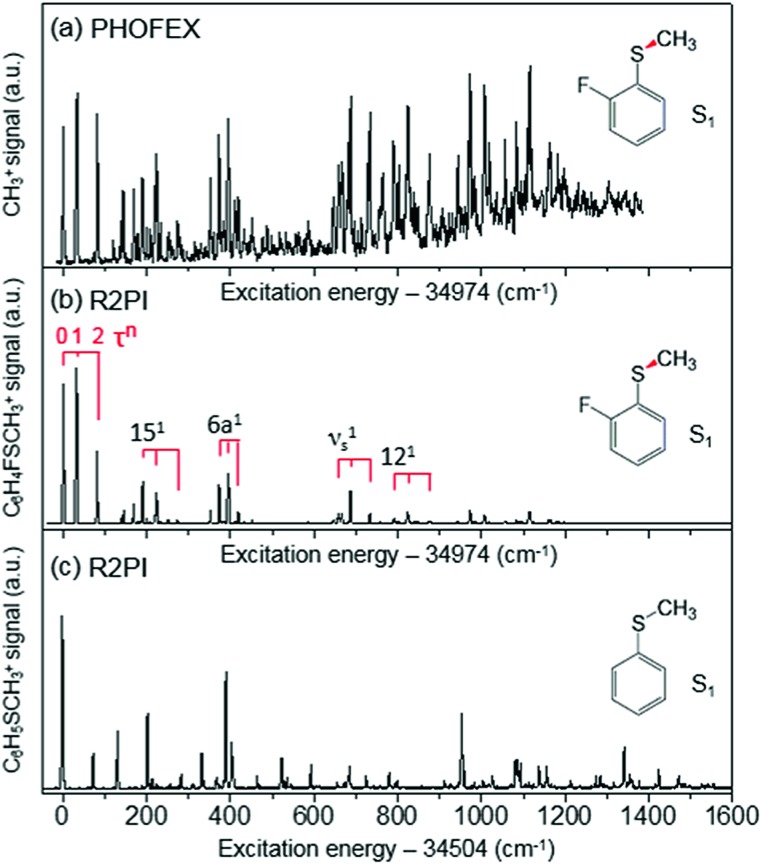
(a) PHOFEX spectrum of 2-fluorothioanisole taken by monitoring the yield of ·CH_3_ (*v* = 0) fragments as a function of the excitation energy. The excitation laser was loosely focused for the PHOFEX spectrum. The R2PI spectra of (b) 2-fluorothioanisole and (c) thioanisole from ref. 37 for comparison.

In the ground state of 2-fluorothioanisole, the *trans*-planar structure is predicted to be more stable than *gauche* by 170 or 150 cm^–1^ according to B3LYP or MP2 calculations with a basis set of 6-311++G(3df,3pd), respectively. NBO analysis also supports this, since the delocalization energy of the *trans*-planar structure is predicted to be ∼6.7 kcal mol^–1^ more stable compared to that of *gauche*. The preferential population of the *trans*-planar conformer of 2-fluorothioanisole was also experimentally reported in the solution phase.^60^ We carried out UV–UV depletion spectroscopy to confirm that there was only one conformational species in the supersonic jet (see the ESI, Fig. S1†), leading us to conclude that the molecular beam of 2-fluorothioanisole adopts a *trans*-planar geometry as the minimum energy structure in the ground state. The R2PI spectrum of 2-fluorothioanisole in the jet (Fig. 2(b)) was found to be quite different from that of thioanisole (Fig. 2(c)) in terms of its overall pattern. Namely, in the case of thioanisole, as the molecule adopts a planar geometry in both the S_0_ and S_1_ states, the S_1_–S_0_ spectral band origin is the most prominent.^37^,^61^–^64^ For 2-fluorothioanisole, however, it was found that the S_1_ minimum-energy structure becomes nonplanar, as clearly manifested by the strongly observed low vibrational frequency bands at 32 and 82 cm^–1^ above the S_1_ origin (34 974 cm^–1^). The most probable vibrational mode for these low-frequency bands is the dihedral torsion of the S–CH_3_ bond with respect to the plane of the benzene moiety. Eigenvalue calculations using a double-well potential energy function of *V*(*x*) = 1/2*kx*^2^ + *A* exp(–*ax*^2^) reproduced the experimental frequencies and intensities very well when the torsional potential barrier height was ∼40 cm^–1^. Here, *V* is the potential energy along the S–CH_3_ dihedral torsional mode (*x*) while *k*, *A*, and a are parameters determining the detailed shape of the double-well potential (see the ESI, Fig. S2†). The progression of the S–CH_3_ out-of-plane torsional mode was found to be actively combined with other in-plane vibrational modes in the R2PI spectrum, confirming that the largest geometrical change upon the S_1_–S_0_ excitation occurs along the torsional angle of the S–CH_3_ bond.

**Fig. 3:**
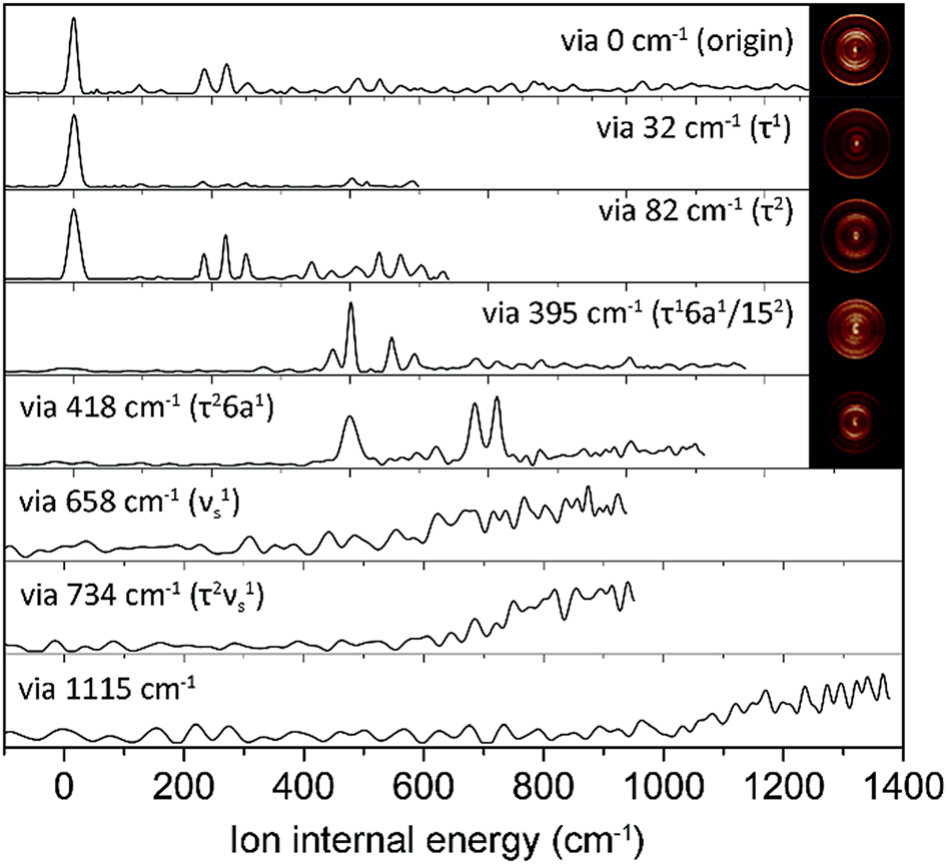
SEVI spectra of 2-fluorothioanisole *via* selected S_1_ intermediate vibronic levels along with assignments in parentheses. Representative photoelectron images are also shown in the inset.

In order to assign S_1_ vibronic bands and obtain information about IVR, we carried out slow-electron velocity-map imaging (SEVI) spectroscopy, and mode assignments are summarized in Table S1.† According to the propensity rule of Δ*v* = 0 applied when the molecular structure is little changed upon photoionization, it is mostly straightforward to assign many S_1_ vibronic bands from the D_0_ modes standing out in the corresponding individual (1 + 1′) SEVI spectra. It is noteworthy that only the cationic origin band is strongly observed in the SEVI spectra taken *via* 32 or 82 cm^–1^, Fig. 3. The same spectral behavior was observed for other molecular systems undergoing large structural change upon ionization.^65^,^66^ Vibrational bands combined with one quantum of out-of-plane τ^+^ (torsional mode) and 10b^+^ modes were persistently observed in the SEVI spectra, indicating that the D_0_–S_1_ transition is accompanied by symmetry-breaking, caused by the nonplanar-to-planar structural change. It is evident that IVR among S_1_/S_2_ vibronic manifolds becomes quite significant at least from the S_1_ internal energy of 658 cm^–1^, as manifested in the diffuse featureless SEVI spectra taken *via* S_1_ intermediate states of the internal energy equal to or higher than 658 cm^–1^ (Fig. 3).^67^ The IVR dynamics in the S_1_/S_2_ coupling regime is intriguing by itself and provides invaluable information, as it results not only from the density of the S_1_ vibrational states but also from the S_1_/S_2_ vibronic coupling, which is strongly influenced by the energetic structure of the S_1_/S_2_ conical intersection seam in multidimensional nuclear configurational space. The zero^th^-order mode character would be no longer guaranteed for vibronic modes observed at S_1_ internal energies higher than 658 cm^–1^. In SEVI spectrum for the S_1_ internal energy of 418 cm^–1^, the sign of IVR onset is observed, indicating that the S_1_/S_2_ coupling already starts to play a major dynamic role in the low internal energy region.

**Fig. 4:**
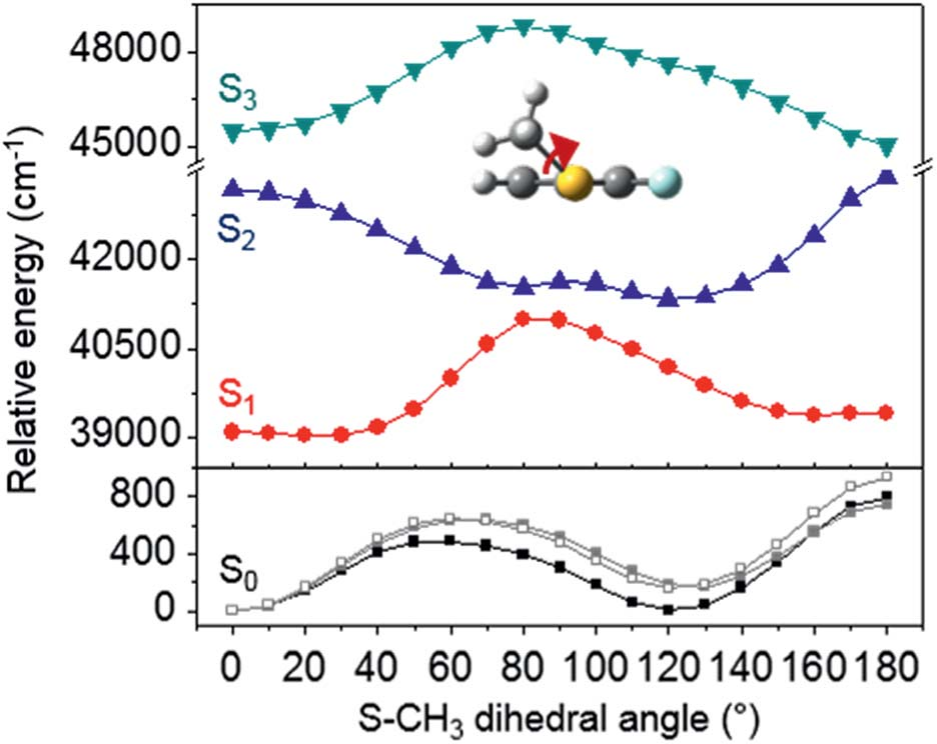
The four-lowest singlet potential energy curves plotted *versus* the S–CH_3_ dihedral angle of S_0_ (black), S_1_ (red), S_2_ (blue) and S_3_ (cyan) states. The excited states were calculated using EOM-CCSD/6-311++G(3df,3pd). The relaxed potential energy curves in S_0_ were obtained using the B3LYP (grey filled square) and MP2 (grey empty square) methods with a 6-311++G(3df,3pd) basis set.

The *ab initio* calculated structures of 2-fluorothioanisole in the four-lowest singlet electronic states in Fig. 4 are qualitatively consistent with the spectroscopic results. Our EOM-CCSD calculation with a basis set of 6-311++G(3df,3pd) predicts the S_1_ minimum structure of the torsional angle of 20°. Even though all of our calculations using TD-DFT, CIS, RICC2, or CASSCF gave the optimized nonplanar S_1_ structure as the global minimum (Fig. S5 in the ESI†), the Franck–Condon analysis based on these calculations did not reproduce the intensity pattern in the experiment. The constrained DFT optimization process, freezing S and F atoms as well as all carbon atoms of the benzene moiety on the molecular plane, gives rise to the S_1_ minimum structure, which matches the experiment quite well at the torsional angle of 12.5° (see the ESI, Fig. S3†), even though the corresponding calculated structure gives two imaginary frequencies.

The S_1_ structural change induced by the simple H/F chemical substitution on the *ortho*-position of thioanisole is expected to have a substantial influence on the nonadiabatic dynamics in the S–CH_3_ bond cleavage reaction, since the S–CH_3_ dihedral torsional mode is parallel with the coupling vectors of both the S_1_/S_2_ and S_0_/S_2_ conical intersections.^38^,^39^ The PHOFEX spectrum obtained by monitoring the ·CH_3_ (*v* = 0) fragment yield as a function of the S_1_–S_0_ excitation energy of 2-fluorothioanisole is quite different from the R2PI spectrum. As shown in Fig. 2(a), the PHOFEX spectrum shows a continuous background signal that starts to grow from ∼400 ± 100 cm^–1^ above the S_1_ origin. It gradually increases and persists until the S_1_ internal energy of ∼1400 cm^–1^, indicating that the quantum yield of the ·CH_3_ (*v* = 0) fragment increases while the R2PI signal diminishes sharply with increasing internal energy. This feature of the PHOFEX spectrum may represent the behaviours of resonances (peaks) in the continuum (background signal) of the S_1_ 2-fluorothioanisole. The different trends in the R2PI and PHOFEX signals in the high energy region may also result from a decrease in S_1_ lifetime with increasing energy.

**Fig. 5:**
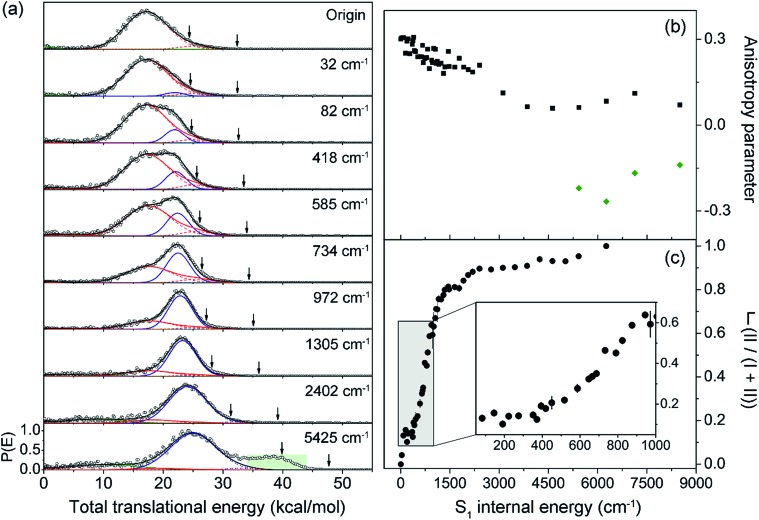
(a) Total translational energy distributions obtained from velocity-map ion images of ·CH_3_ fragment of 2-fluorothioanisole at pump energies from origin to an S_1_ internal energy of 5425 cm^–1^ (black circles). Total distributions are deconvoluted to the low (red lines) and high (blue lines) translational energy channels, both containing Ã and X[combining tilde] states of fragments. Sums of all the fitted lines including minor statistical background most likely originated from multiphoton dissociative ionization (olive lines) are plotted as black lines. The arrows indicate the maximum possible translational energies for channels producing Ã and X[combining tilde] state radicals. (b) Anisotropy parameter (*β*) is plotted as a function of excitation energy (black squares). The *β* values averaged over the range covering the peak at ∼40 kcal mol^–1^ (green shaded area in (a)) are depicted as green diamonds. (c) The fraction (*Γ*) of high translational energy channel (II) in the total distribution as a function of the excitation energy (black filled circles). See the ESI† for more details.

Next, the angular and total translational energy distributions of fragments obtained by the VMI method were analysed thoroughly for all of the S_1_/S_2_ vibronic bands of 2-fluorothioanisole as shown in Fig. 5. The upper limit for the S–CH_3_ bond dissociation energy was set to be 67.6 kcal mol^–1^, which is slightly lower than the 70.8 (ref. 37) (70.5 (ref. 38)) kcal mol^–1^ of thioanisole. At the S_1_ origin, the product translational energy distribution shows a single Gaussian-shaped function peaked at ∼17.2 kcal mol^–1^ although a tiny contribution of the nonadiabatic channel leading to the C_6_H_4_FS·(X[combining tilde]) + ·CH_3_ fragments is responsible for the slightly asymmetric shape in the high energy region. The X[combining tilde]/Ã branching ratio is estimated to be ∼0.06 for 2-fluorothioanisole and this is very similar to that found for thioanisole at the S_1_ origin.^37^ The obvious bimodal shape of the translational energy distribution is already apparent when the S_1_ internal energy is only 82 cm^–1^. Even though one may be tempted to attribute this shoulder-like feature in the high-energy region to the one contributed from the nonadiabatic channel giving C_6_H_4_FS·(X[combining tilde]), this could not be the case simply because the energetic difference between the two deconvoluted Gaussian-shaped distributions (∼5 kcal mol^–1^) is much smaller than the energetic gap between C_6_H_4_FS·(Ã) and C_6_H_4_FS·(X[combining tilde]) which is ∼8.0 kcal mol^–1^ (2800 cm^–1^) estimated from the previous studies of 2-fluorothiophenol.^31^,^35^ This value is slightly smaller than ∼8.6 kcal mol^–1^ (3000 cm^–1^) measured for that of C_6_H_5_S·.^68^ The highest kinetic energy of the fragment is even below the maximum available translational energy of C_6_H_4_FS·(Ã), Fig. 5, and thus this naturally indicates that a new reaction channel (II) mostly giving C_6_H_4_FS·(Ã) with completely different dynamic outputs from those of the existing adiabatic channel (I) starts to be opened (Fig. 1). The growing part of the translational energy distribution in the high energy region shows a higher average translational energy than that in the relatively low energy region, meaning that the kinetic energy release in channel II is larger and the internal energy excitation of the fragments is smaller. Reaction channel II is expected to be much faster compared to channel I, leading to narrower distributions of both the internal and translational energies of its fragments, compared to those from I. This is because in channel II there is not enough time for the large volume of phase space to be explored prior to coupling to the repulsive S_2_ state. The anisotropy parameter (*β*) averaged over the entire energy distribution (15 kcal mol^–1^) was found to be slightly positive (*β* ∼ 0.2) for all vibronic bands in the 0–2400 cm^–1^ region above the S_1_ origin as shown in Fig. 5(b). Unfortunately, no information about the transition dipole moments involved in the S_1_/S_2_–S_0_ excitation could be inferred from the anisotropy parameters because the excited-state lifetime of 2-fluorothioanisole is not expected to be faster than the rotational period of the entire molecule.^45^

Quite intriguingly, the branching ratio of channel II with respect to channel I increased very rapidly as the excitation energy increased. Firstly, it shows a step-like structure. The yield of channel II (*Γ* ≡ II/(I + II)) in Fig. 5(c) shows an abrupt increase from 0.04 at 32 cm^–1^ to 0.13 at 82 cm^–1^ (τ^2^). And then it remains more or less constant while it fluctuates within the 0.10–0.16 range up to the excitation energy of 400 cm^–1^. Incidentally, *Γ* shows slight drops at the 191 and 373 cm^–1^ bands, associated with the 15 and 6a in-plane vibrational modes, respectively. This in turn indicates that *Γ* increases when out-of-plane modes are involved in the excited state. Thereafter, *Γ* increases dramatically to ∼0.8 at ∼1200 cm^–1^. The dominance of channel II with the increase in excitation energy in this energy region could be attributed to an increase of the accessibility of corresponding quantum states to the upper-lying planar conical intersection, probably because this region includes τ progressions combined with symmetric and/or asymmetric C–S–CH_3_ stretching modes (ν_s_ and/or 7a, respectively) which are parallel to the gradient vector of the conical intersection (Table 1). This could be reflected in the slight but evident enhancement of *Γ* at ∼734 cm^–1^, Fig. 5(c), which is tentatively assigned to τ^2^ν_s_. The low translational energy component due to channel I almost completely disappears above ∼2400 cm^–1^. When the excitation energy is higher than 4600 cm^–1^ above the S_1_ origin, another electronic transition (S_3_–S_0_) comes in and gives rise to a significant contribution of the nonadiabatic channel, giving away the C_6_H_4_FS·(X[combining tilde]) fragment. The reaction rates at such high energies should be faster than the rotational period of the whole molecule, showing a negative anisotropy parameter of ∼–0.2. The dynamics at such high excitation energies are expected to be quite different and subject to further investigation.

**Table 1:** Planar minimum-energy conical intersection (MECI) and nonplanar quasi-degenerate S_1_/S_2_ crossing point calculated using SA4-CASSCF(12,11)/6-311++G(3df,3pd)

	Planar CI	Nonplanar quasi-seam
Gradient difference vector	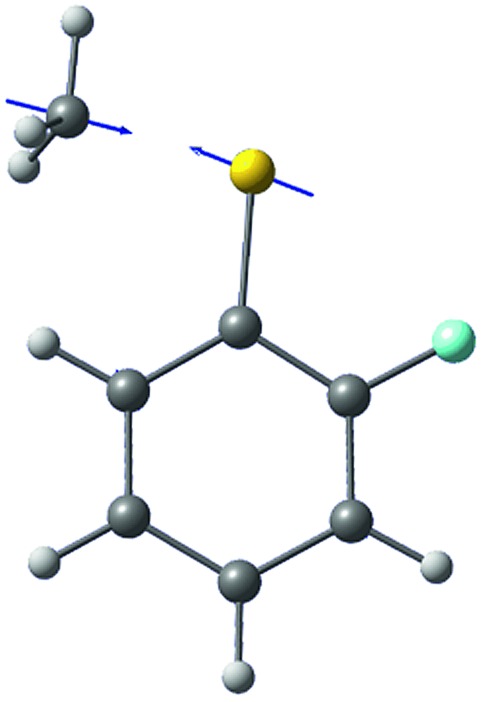	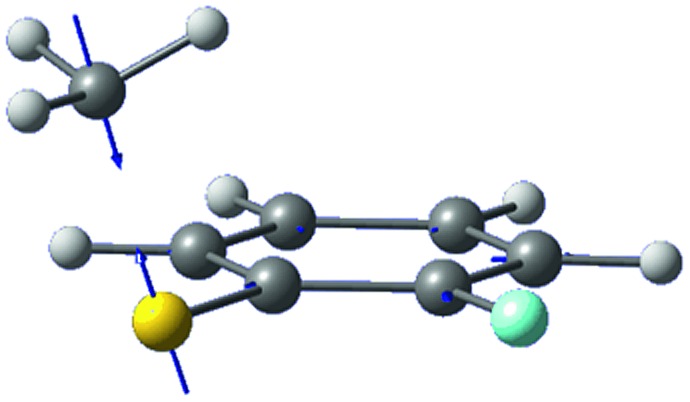
Derivative coupling vector	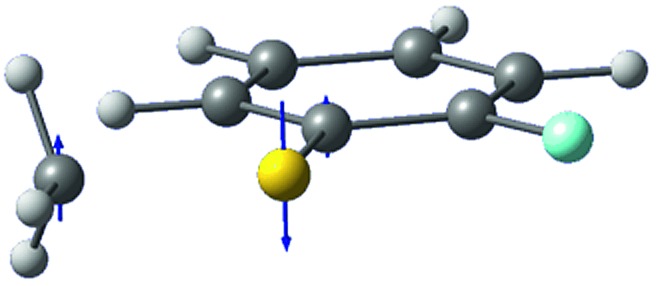	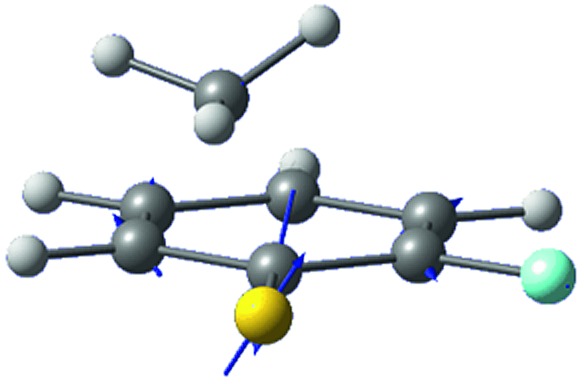
*R*_S-CH_3__ (Å)	2.13	2.11
S–CH_3_ dihedral angle	0°	–79°
Δ*E*_1min_CI_ (cm^–1^)^*a*^	1518	827
Δ*E*_1_2_ (cm^–1^)^*b*^	2	164

^*a*^Energy difference between S_1_ minimum (vertical) and the CI.

^*b*^Energy difference between S_1_ and S_2_ states at the CI.

The experimental fact that two distinct reaction channels exist, giving completely separable product state distributions indicates that the reactive fluxes prepared at resonances in the continuum prepared in the strong coupling regime of the bound (S_1_) and unbound (S_2_) states of 2-fluorothioanisole bifurcate into two totally different reaction pathways, as in Fig. 1. Our recent time-resolved work on thioanisole clearly demonstrated that a reactive flux prepared in the proximity of the conical intersection bifurcates into either an adiabatic or nonadiabatic reaction pathway with distinct reaction times, as well as different energy disposals and nonadiabatic transition probabilities.^45^ The nonadiabatic process was measured to be much faster than the adiabatic pathway. That situation is very similar to the one here with 2-fluorothioanisole, except that both the adiabatic and nonadiabatic parts of the initially-prepared reactive flux in the Franck–Condon region mostly end up with the adiabatic channel (C_6_H_4_FS·(Ã)) in the asymptotic limit. This is because 2-fluorothioanisole adopts a nonplanar geometry in both S_1_ and S_2_ as shown in Fig. 4. This theoretical prediction strongly suggests that the reactive flux riding on the repulsive S_2_ state will experience strong torque along the S–CH_3_ torsional coordinate as the S–CH_3_ bond is elongated. The nonplanarity of 2-fluorothioanisole in S_2_ would make the passage of the reactive flux remote from the S_0_/S_2_ conical intersection located on the planar geometry, providing a perfect explanation for the nearly complete absence of the C_6_H_4_FS·(X[combining tilde]) + ·CH_3_ fragmentation channel.

Interestingly, in Fig. 4, the potential energy curves of the S_1_ and S_2_ state are close at the S–CH_3_ dihedral angle of ∼80°. The conical intersection optimization shows that a quasi-degenerate S_1_/S_2_ surface crossing seam, where the energy gap between the lower and upper adiabats is small, exists in the nonplanar geometry, although the S_1_/S_2_ minimum-energy conical intersection (MECI) adopts a planar geometry, Table 1. According to our CASSCF calculations, a quasi-degenerate S_1_/S_2_ crossing seam is located ∼700 cm^–1^ below the planar MECI and ∼830 cm^–1^ above the S_1_ minimum. The calculated energetic position of nonplanar quasi-seam with respect to the S_1_ minimum is actually very low considering the fact that CASSCF values are often overestimated. The nonplanar quasi-seam adopts a geometry where the S–CH_3_ bond length is 2.11 Å and a S–CH_3_ dihedral angle of – 79°. Since it is energetically located at a much lower position than the planar MECI, at least the reactive flux prepared in the low S_1_ internal energy region may have a chance to explore the phase space around the nonplanar S_1_/S_2_ quasi-seam, especially since the S–CH_3_ torsional mode is strongly activated in the S_1_–S_0_ optical excitation. Actually, a similar conceptual approach may underlie the previous findings that symmetry breaking in the photoexcitation of catechol,^19^,^20^ 2-aminophenol,^22^ or 2-fluorophenol–(NH_3_) cluster^16^ is responsible for the shortening of excited-state lifetimes.

It seems that strong coupling of the bound (S_1_) and unbound (S_2_) states in the nonplanar geometry transforms the initially-prepared quantum state immediately into a mixture of contributions from the upper and lower adiabats. Since the quasi-degenerate S_1_/S_2_ crossing seam developed in the nonplanar nuclear configuration is expected to be located just a few hundreds of cm^–1^ above the S_1_ minimum, there might be a significant chance for the reactive flux confined in the upper adiabat to leak into the repulsive S_2_ state through the nonplanar quasi-seam. Since the out-of-plane S–CH_3_ torsional mode is highly excited in S_1_, the S_1_/S_2_ coupling should be very efficient over the wide range of nuclear configurations. Similar to the case of thioanisole, it seems straightforward to identify the origins of the high and low translational energy components in the total translational energy distributions of products from 2-fluorothioanisole (*vide supra*). Obviously, the reactive flux initially confined in the upper adiabat gives rise to fragments less excited along the vibrational degrees of freedom orthogonal to the reaction coordinate. This should produce a larger kinetic energy release, giving the higher and narrower translational energy distribution of the fragments. Nonadiabatic leakage from the upper adiabat through either the S_1_/S_2_ conical intersection or nonplanar quasi-seam is followed by the prompt S–CH_3_ bond breakage on the repulsive S_2_ state (channel II). In the meantime, the relatively low component of the translational energy distribution results from exploration of the reactive flux sticking to the lower adiabatic potential energy surfaces. Here, in order to overcome the adiabatic barrier, whose top corresponds to the saddle point along the S–CH_3_ elongation coordinate, the reactive flux explores the large volume of phase space prior to reaching the critical configurations necessary for riding on S_2_ (channel I). Therefore, this time-consuming exploration gives rise to internally hot fragments, giving naturally low and broad translational energy product distributions. As the excitation energy increases, channel II becomes dominant with the aid of IVR, resulting in the broader translational energy distributions observed at higher excitation energies.

## Conclusions

Herein, we report that the nonadiabatic bifurcation phenomenon is robust when the quantum state is prepared at resonances in the continuum generated by the strong coupling of bound and unbound states in the Franck–Condon region along the reaction coordinate. Our observation clearly demonstrates that the reactive flux in the strong bound–unbound vibronic coupling regime can be described as the quantum state of mixed characteristics belonging to either the upper or lower adiabatic states. The vibronic state mixing includes both IVR and electronic coupling beyond the Born–Oppenheimer approximation, and we determined that both Herzberg type-I (electronic) and type-II (vibrational) predissociation mechanisms coexist and proceed in a competitive way. Competition between the two mechanisms is very sensitive to the location of the reactive flux with respect to the Franck–Condon and/or coupling region in the multidimensional nuclear configuration. In 2-fluorothioanisole, it was possible to investigate the dynamics taking place in the nonplanar nuclear configurational space thanks to the particular fact that the molecule undergoes a planar-to-nonplanar structural change upon the first electronic excitation. Chemical substitution turns out to be a quite valuable tool for exploring dynamics in a variety of nuclear configurational spaces. The excitation energy dependent structures of fractions of two competing channels should represent vibronic coupling matrix elements in the expression of Fermi's golden rule.^69^ Full dimensional calculations of the potential energy surfaces are desirable to arrive at a quantitative explanation of the experiment in the near future.

## Conflicts of interest

There are no conflicts to declare.

## Supplementary Material

Supplementary information
