# Supplementary material for: Experimental observation of nonadiabatic bifurcation dynamics at resonances in the continuum
Source: Chem Sci. 2019 Jan 4;10(8):2404–12. doi: 10.1039/c8sc04859b (PMC6385646; doi:10.1039/c8sc04859b)
Supplement: Supplementary file 1 [file SC-010-C8SC04859B-s001.pdf]

Electronic Supplementary Material (ESI) for Chemical Science.
This journal is © The Royal Society of Chemistry 2019

Supplementary Information

Experimental observation of nonadiabatic bifurcation dynamics at resonances in the continuum

Jean Sun Lim,† Hyun Sik You,†‡ So-Yeon Kim§ and Sang Kyu Kim*

Department of Chemistry, KAIST, Daejeon 34141, Republic of Korea

Table of Contents

1. UV-UV depletion spectroscopy of 2-fluorothioanisole

2. Double-well potential for S_1 state of 2-fluorothioanisole

3. Franck-Condon simulation

4. Assignments of R2PI and SEVI spectra, and calculated vibrational frequencies in S_0 and D_0 states

5. Global minimum geometries in S_1 state by several *ab initio* calculations

6. Velocity-map ion images and deconvolution of total translational energy distributions

7. UV absorption spectra in hexane

1. UV-UV depletion spectroscopy of 2-fluorothioanisole

UV-UV depletion spectroscopy was performed to check that vibronic transitions start from the same zero-point level of the ground state of the one isomer. The burning laser was scanned while the wavelength of the probe laser was fixed at the band origin with a time delay of 100 ns.

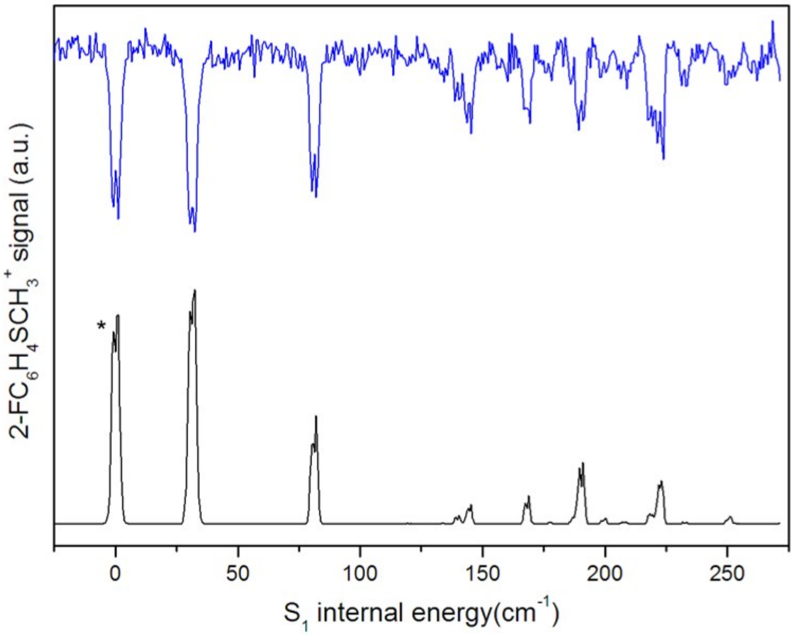

Figure S1. UV-UV depletion spectrum (blue line) compared to R2PI spectrum (black line) of 2-fluorothioanisole. The asterisk indicates the fixed probing transition.

2. Double-well potential for S_1 state of 2-fluorothioanisole

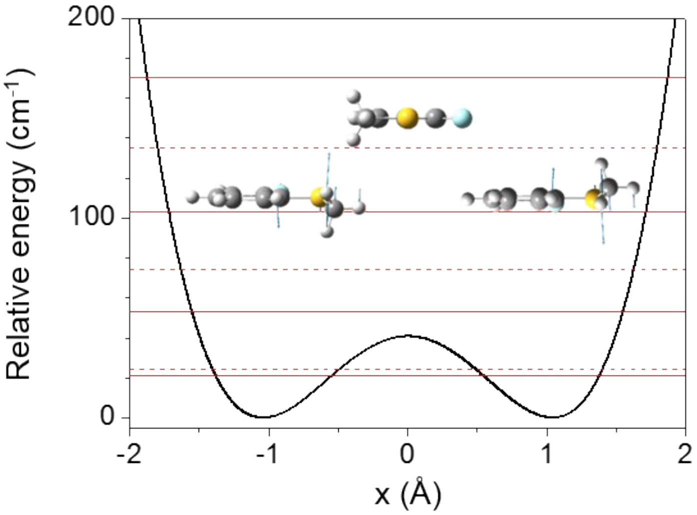

Figure S2. Gaussian double-well potential along S-CH_3 torsional mode. Parameters A = 3358 cm^{-1} and a = 0.1519 $Å^{-2}$ with a barrier of 40 cm^{-1} were obtained from the fitting of origin (0 cm^{-1}), τ^1 (32 cm^{-1}), and τ^2 (82 cm^{-1}) bands in R2PI spectrum. (k = 0.0172 mdyne/Å) The corresponding ratio of the Franck-Condon factors for these bands is 1:1.7:0.3. The red solid and dashed lines are symmetrically allowed and forbidden transition levels, respectively. The eigenvalues and eigenfunctions for the potential was obtained using Wavepacket 4.7.3[1]. The time-independent Schrődinger equation was solved by the Fourier grid Hamiltonian method. The parameters of the reduced mass and the force constants were taken from the constrained optimization (see Figure S3).

3. Franck-Condon simulation

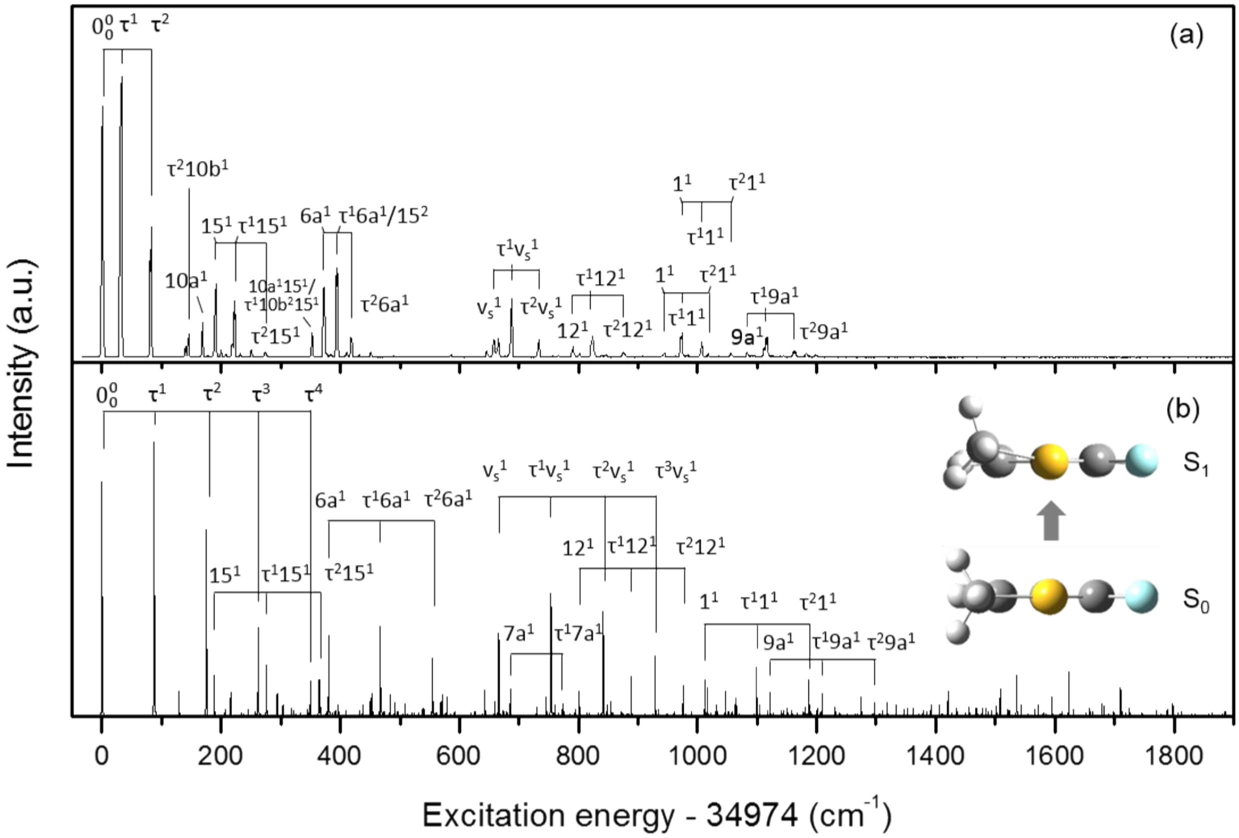

Figure S3. (a) R2PI spectrum and (b) Franck-Condon simulation of 2-fluorothioanisole. The optimized geometry of S_1 state in the inset was obtained by freezing six geometry parameters using TD-B3LYP[2]/6-311++G(d,p). The S-CH_3 dihedral angle is fixed at 12.5°, and all the atoms except for the hydrogens and methyl group are in-plane. The Franck-Condon simulation was performed with this S_1 geometry using FCLabII.[3, 4] The two imaginary frequencies (-87 and -146 cm^{-1}) were converted to the positive ones for the simulation.

4. Assignments of R2PI and SEVI spectra, and calculated vibrational frequencies in S_0 and D_0 states

Table S1. Assignment of S_1 vibronic bands of 2-fluorothioanisole based on SEVI spectra and Franck-Condon simulation.

Energy (cm^{-1})	S_1 internal energy (cm^{-1})	Assignment
34974	0	0_0^0
35006	32	τ^1
35056	82	τ^2
35119	145	$\tau^2 10b^1$
35143	169	$10a^1$
35165	191	15^1
35197	223	$\tau^1 15^1$
35248	274[a]	$\tau^2 15^1$
35327	353	$10a^1 15^1/\tau^1 10b^2 15^1$
35347	373	$6a^1$
35369	395	$\tau^1 6a^1/15^2$
35392	418	$\tau^2 6a^1$
35632	658[b]	ν_s^1
35672	666[b]	ν_s^1
35662	688[b]	$\tau^1 \nu_s^1$
35708	734[b]	$\tau^2 \nu_s^1$
35765	791[c]	12^1
35798	824[c]	$\tau^1 12^1$
35850	876[c]	$\tau^2 12^1$
35918	944[a]	1^1
35947	973[a]	$\tau^1 1^1$

35992	1018[a]	$\tau^2 1^1$
36057	1083[a]	$9a^1$
36090	1116[a]	$\tau^1 9a^1$
36137	1163[a]	$\tau^2 9a^1$

[a] These assignments are based on the Franck-Condon simulation.

[b] Both 658 and 666 cm^{-1} S_1 bands have ν_s mode characters in the SEVI spectra, and corresponding assignments are tentatively given by considering the spectral pattern of R2PI spectrum.

[c] 824 cm^{-1} S_1 band has a mode character of 12 and corresponding assignments are tentatively given by considering the spectral pattern of R2PI spectrum.

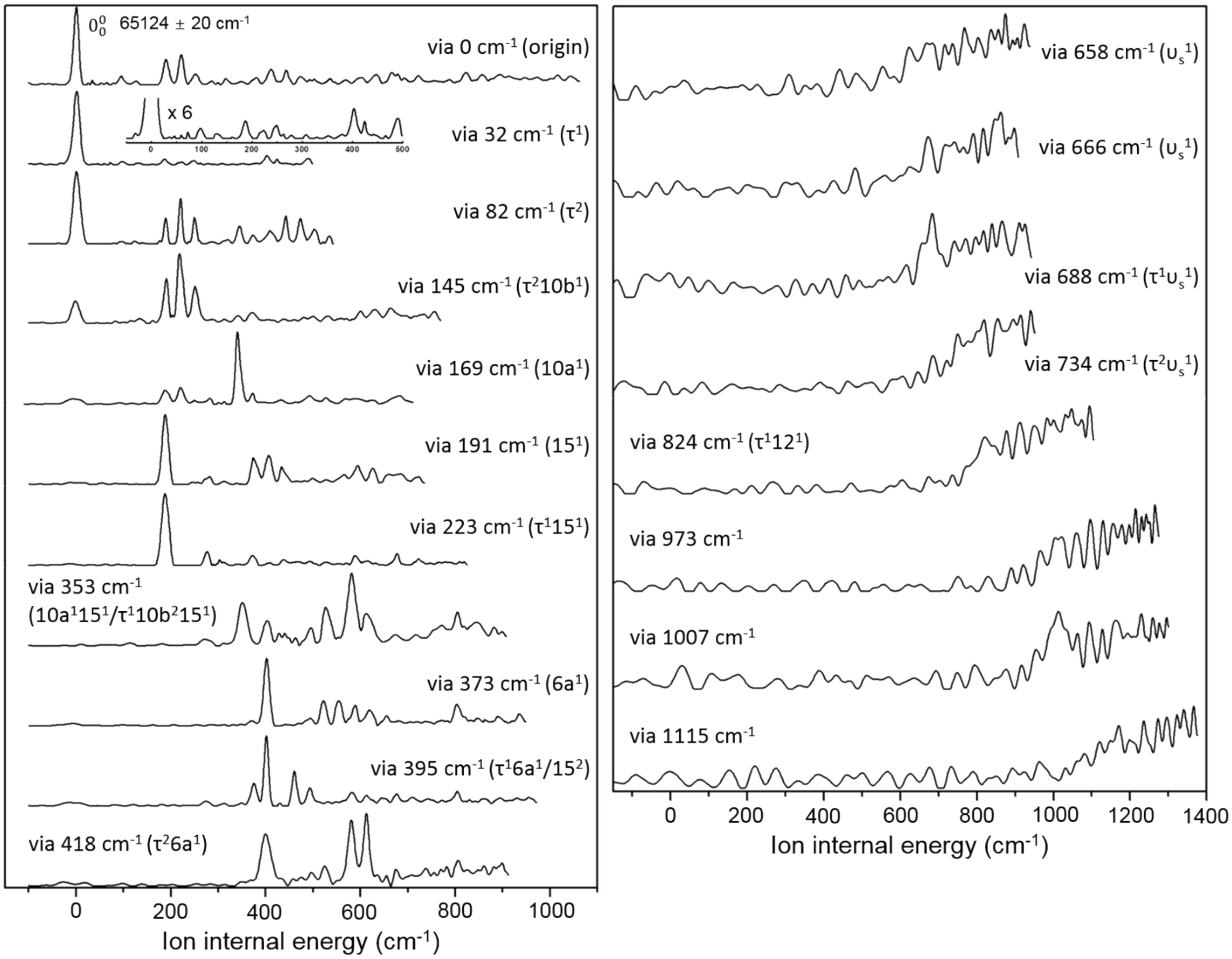

Figure S4. SEVI spectra of 2-fluorothioanisole via S_1 intermediate vibronic levels indicated along with assignments in parentheses.

Table S2. Experimental and calculated vibrational frequencies (cm^{-1}) in S_0 and D_0 states of 2-fluorothioanisole. The S_0 and D_0 frequencies were obtained using B3LYP/6-311++G(3df,3pd) level.

Mode[a]	Symmetry	S_0	S_1	D_0	
		Calc.	Exp.[b]	Calc.	Exp.[c]
τ	a”	57	32	95	97
10b	a”	149	63	126	127
τCH_3	a”	233		178	
15	a’	183	191	184	188
10a	a”	275	169	254	250
β_s	a’	262		272	
6a	a’	400	373	406	402
16b	a”	452		440	
16a	a”	553		495	496
20a	a’	492		500	
6b	a’	559		543	
ν_s	a’	683	658	680	684
7a	a’	725		715	
4	a”	717		722	
11	a”	764		778	
12	a’	829	791	831	821
17b	a”	863		890	
$\gamma_s CH_3$	a”	969		935	
$\beta_{as} CH_3$	a’	985		990	
17a	a”	950		993	
5	a”	991		1023	
1	a’	1056		1032	
19b	a’	1092		1106	
19a	a’	1142		1156	
9b	a’	1185		1204	
9a	a’	1243		1244	
3	a’	1289		1311	

14	a’	1327	1350
$\beta_s CH_3$	a’	1357	1360
$\beta_{as} CH_2$	a’	1486	1452
$\gamma_{as} CH_3$	a”	1469	1459
18a	a’	1476	1466
18b	a’	1507	1475
8b	a’	1607	1546
8a	a’	1633	1611
$\nu_s CH_3$	a’	3039	3050
$\nu_{as} CH_2$	a”	3120	3146
$\nu_{as} CH_3$	a’	3131	3154
13	a’	3178	3200
7b	a’	3193	3211
20b	a’	3205	3219
2	a’	3210	3229

[a] The torsion vibration and stretching vibration are denoted by τ and ν, respectively. The bending mode in which the vibrating atoms preserve a well-defined plane is designated by β. The perpendicular bending vibration with respect to such a plane is denoted by γ. The symmetric or asymmetric character of the vibration is indicated by a subscript. Of the bending modes of a CH_n group, β_s designates the scissoring vibration and β_{as}, γ_s, and γ_a denote the rocking, wagging, and twisting modes, respectively. Normal modes are labeled according to Ref. [5].

[b] The experimental values are observed from R2PI spectrum.

[c] The experimental values are observed from SEVI spectra.

Table S3. Assignments of vibrational frequencies of 2-fluorothioanisole in D_0 state appeared in (1+1’) SEVI spectra via S_1 intermediate vibronic states.

Ion internal energy (cm^{-1})	Assignment	Ion internal energy (cm^{-1})	Assignment
(a) via S_1 0^0_0 (0 cm^{-1})			
0[a]	0^0_0	96	τ^1
127	$10b^1$	189[a]	τ^2 / 15^1

222	$\tau^1 10b^1$	253	$10a^1/10b^2$
286	τ^3 / $\tau^1 15^1$	316	$\tau^2 10b^1$ / $10b^1 15^1$
349	$\tau^1 10a^1$ / $\tau^1 10b^2$	380	τ^4 / $10b^1 10a^1$ / $\tau^2 15^1$ / 15^2 / $10b^3$
411	$6a^1$ / $\tau^1 10b^1 15^1$ / $\tau^3 10b^1$	443	$\tau^2 10b^2$ / $\tau^2 10a^1$ / $10b^2 15^1$ / $10a^1 15^1$
473	$\tau^1 10b^3$ / $\tau^1 10b^1 10a^1$ / $\tau^3 15^1$ / $\tau^1 15^2$ / $\tau^1 15^2$	503	$\tau^1 6a^1$ / $\tau^2 10b^1 15^1$ / $10a^2$ / $10b^4$ / $10a^1 10b^2$ / $10b^1 15^2$
536	$10b^1 6a^1$ / $\tau^1 10b^2 15^1$ / $\tau^1 10a^1 15^1$ / $\tau^3 10b^2$	569	$10b^1 10a^1 15^1$ / $\tau^2 10b^1 10a^1$ / $10b^3 15^1$ / $\tau^2 10b^3$ / $\tau^2 15^2$
602	$\tau^3 10b^1 15^1$ / $\tau^1 10b^1 15^2$ / $\tau^2 6a^1$ / $\tau^1 10a^2$ / $\tau^1 10a^1 10b^2$/ $\tau^1 10b^4$	633	$\tau^2 10b^2 15^1$ / $10b^2 15^2$ / $\tau^2 10a^1 15^1$ / $\tau^1 10b^1 6a^1$ / $10b^1 10a^2$ / $10a^1 10b^3$
666	$10b^2 6a^1$ / $\tau^1 6b^1$ / $\tau^3 15^2$ / $\tau^1 10b^3 15^1$ / $\tau^3 10b^1 10a^1$	679	ν_s^1 / $10a^2 15^1$ / $10b^4 15^1$ / $\tau^1 15^1 6a^1$ / $\tau^2 10b^1 15^2$ / $\tau^3 6a^1$
693	$\tau^2 10b^2 10a^1$ / $10b^2 10a^1 15^1$ / $\tau^3 6a^1$ / $\tau^1 15^1 6a^1$ / $\tau^2 10a^2$ / $\tau^2 10b^1 15^2$	722	$\tau^3 10a^1 15^1$ / $\tau^1 10a^1 15^2$ / $\tau^3 10b^2 15^1$ / $\tau^1 10b^2 15^2$ / $\tau^2 10b^1 6a^1$ / $10b^1 15^1 6a^1$ / $\tau^1 10b^1 10a^2$ / $\tau^1 10b^3 10a^1$
823	$10b^3 10a^1 15^1$ / $\tau^2 10b^2 15^2$ / $\tau^3 10b^1 6a^1$ / $\tau^2 10b^1 10a^2$ / $\tau^2 10b^3 10a^1$ / 12^1	856	$\tau^2 10b^2 6a^1$ / $\tau^1 10b^3 15^2$ / $\tau^2 10a^1 6a^1$ / $\tau^1 10b^2 10a^2$
914	$\tau^3 10b^3 10a^1$ / $\tau^3 10b^2 15^2$ /	973	$\tau^1 10b^3 10a^2$ / $\tau^2 10b^3 6a^1$ /

	$\tau^3 10b^1 10a^2$ / $\tau^1 12^1$		$\tau^1 10b^1 10a^3$
1016	$\tau^4 10b^2 15^2$ / $\tau^2 12^1$		
(b) via S_1 τ^1 (32 cm^{-1})			
0[a]	0_0^0	97	τ^1
129	$10b^1$	187	τ^2
224	$\tau^1 10b^1$	249	$10a^1$/$10b^2$
403	$6a^1$	424	$\tau^3 10b^1$
491	$\tau^1 6a^1$ / $10a^2$ / $10b^4$ / $10a^1 10b^2$		
(c) via S_1 τ^2 (82 cm^{-1})			
0[a]	0_0^0	97	τ^1
123	$10b^1$	189[a]	τ^2
220[a]	$\tau^1 10b^1$	250[a]	$10a^1$ / $10b^2$
345	$\tau^1 10a^1$ / $\tau^1 10b^2$	374	τ^4 / $10b^1 10a^1$/ $10b^3$
410	$6a^1$ / $\tau^3 10b^1$	443[a]	$\tau^2 10b^2$ / $\tau^2 10a^1$
473[a]	$\tau^1 10b^3$ / $\tau^1 10b^1 10a^1$	503	$10a^2$ / $10b^4$ / $10a^1 10b^2$ / $\tau^1 6a^1$
(d) via S_1 $\tau^2 10b^1$ (145 cm^{-1})			
0[a]	0_0^0	100	τ^1
133	$10b^1$	191[a]	τ^2
218[a]	$\tau^1 10b^1$	251[a]	$10a^1$/$10b^2$
313	$\tau^2 10b^1$	343	$\tau^1 10b^2$ / $\tau^1 10a^1$
372	τ^4 / $10b^1 10a^1$ / $10b^3$		

(e) via S_1 $10a^1$ (169 cm^{-1})			
0	0^0_0	93	τ^1
120	$10b^1$	187[a]	τ^2
220[a]	$\tau^1 10b^1$	250	$10a^1/10b^2$
283	τ^3	340[a]	$\tau^1 10a^1$ / $\tau^1 10b^2$
373	τ^4 / $10b^1 10a^1$ / $10b^3$	432	$\tau^2 10b^2$ / $\tau^2 10a^1$
(f) via S_1 15^1 (191 cm^{-1})			
188[a]	15^1	246	$10a^1/10b^2$
282	$\tau^1 15^1$	313	$10b^1 15^1$
374[a]	$\tau^2 15^1$ / 15^2	407[a]	$\tau^1 10b^1 15^1$ / $6a^1$
433	$10b^2 15^1$ / $10a^1 15^1$	500	$\tau^2 10b^1 15^1$ / $10b^1 15^2$/ $\tau^1 6a^1$
566	$10b^1 10a^1 15^1$ / $10b^3 15^1$	594	$\tau^3 10b^1 15^1$ / $\tau^2 6a^1$
627	$\tau^2 10b^2 15^1$ / $\tau^2 10a^1 15^1$ / $10b^2 15^2$ / $\tau^1 10b^1 6a^1$	660	$10b^2 6a^1$ / $\tau^3 15^2$
685	$10a^2 15^1$ / $10b^4 15^1$ / $\tau^2 10b^1 15^2$ / $\tau^3 6a^1$ / $\tau^1 15^1 6a^1$		
(g) via S_1 $\tau^1 15^1$ (223 cm^{-1})			
187[a]	15^1	277[a]	$\tau^1 15^1$
311	$10b^1 15^1$	372	$\tau^2 15^1$ / 15^2
410	$\tau^1 10b^1 15^1$ / $6a^1$	438	$10b^2 15^1$ / $10a^1 15^1$
469	$\tau^3 15^1$ / $\tau^1 15^2$	494	$\tau^2 10b^1 15^1$ / $\tau^1 6a^1$ / $16a^1$
528	$\tau^1 10a^1 15^1$ / $\tau^1 10b^2 15^1$ / $10b^1 6a^1$	588	$\tau^3 10b^1 15^1$ / $\tau^2 6a^1$ / $\tau^1 10a^2$ / $\tau^1 10b^4$ /

			$\tau^{1}10a^{1}10b^{2}$
678	$10a^{2}15^{1}$ / $10b^{4}15^{1}$ / $\tau^{2}10b^{1}15^{2}$ / $\tau^{3}6a^{1}$ / $\tau^{1}15^{1}6a^{1}$	722	$\tau^{3}10a^{1}15^{1}$ / $\tau^{1}10a^{1}15^{2}$ / $\tau^{1}10b^{2}15^{2}$ / $\tau^{2}10b^{1}6a^{1}$ / $10b^{1}15^{1}6a^{1}$
(h) via S_1 $10a^{1}15^{1}/\tau^{1}10b^{2}15^{1}$ (353 cm^{-1})			
115	$10b^{1}$	180	τ^{2} / 15^{1}
272	$\tau^{1}15^{1}$ / τ^{3}	351[a]	$\tau^{1}10a^{1}$ / $\tau^{1}10b^{2}$
404[a]	$\tau^{1}10b^{1}15^{1}$ / $6a^{1}$	428	$10b^{2}15^{1}$ / $10a^{1}15^{1}$ / $\tau^{2}10b^{2}$ / $\tau^{2}10a^{1}$
441	$10b^{2}15^{1}$ / $\tau^{2}10b^{2}$ / $\tau^{2}10a^{1}$ / $10a^{1}15^{1}$	496	$\tau^{2}10b^{1}15^{1}$ / $10b^{1}15^{2}$ / $\tau^{1}6a^{1}$
526[a]	$\tau^{1}10b^{2}15^{1}$ / $\tau^{1}10a^{1}15^{1}$ / $10b^{1}6a^{1}$	581[a]	$\tau^{3}10b^{1}15^{1}$ / $\tau^{1}10a^{2}$ / $\tau^{1}10b^{4}$ / $\tau^{1}10a^{1}10b^{2}$ / $\tau^{2}6a^{1}$
613[a]	$\tau^{2}10a^{1}15^{1}$ / $\tau^{2}10b^{2}15^{1}$ / $\tau^{1}10b^{1}6a^{1}$ / $10b^{2}15^{2}$	804[a]	$\tau^{2}10b^{2}15^{2}$ / $\tau^{3}10b^{1}6a^{1}$ / $6a^{2}$
(i) via S_1 $6a^{1}$ (373 cm^{-1})			
402[a]	$6a^{1}$ / $\tau^{1}10b^{1}15^{1}$	495	$\tau^{1}6a^{1}$/ $\tau^{2}10b^{1}15^{1}$
522[a]	$10b^{1}6a^{1}$ / $\tau^{1}10b^{2}15^{1}$ / $\tau^{1}10a^{1}15^{1}$	555[a]	$6b^{1}$ / $\tau^{2}15^{2}$
589[a]	$\tau^{2}6a^{1}$ / $\tau^{3}10b^{1}15^{1}$ / $\tau^{1}10a^{2}$ / $\tau^{1}10b^{4}$ / $\tau^{1}10a^{1}10b^{2}$	619	$\tau^{1}10b^{1}6a^{1}$ / $\tau^{2}10a^{1}15^{1}$ / $10b^{2}15^{2}$ / $\tau^{2}10b^{2}15^{1}$
655	$10b^{2}6a^{1}$ / $\tau^{1}6b^{1}$ / $\tau^{3}15^{2}$	803[a]	$6a^{2}$ / $\tau^{3}10b^{1}6a^{1}$ /

			$\tau^{2}10b^{2}15^{2}$ / $10b^{2}6b^{1}$ / $10b^{1}\nu_{s}^{1}$
(j) via S_1 $\tau^{1}6a^{1}$ /15^{2} (395 cm^{-1})			
275	$\tau^{1}15^{1}$	375[a]	$\tau^{2}15^{1}$ / 15^{2}
401[a]	$6a^{1}$ / $\tau^{1}10b^{1}15^{1}$	460[a]	$\tau^{1}15^{2}$ / $\tau^{3}15^{1}$
494[a]	$\tau^{1}6a^{1}$ / $\tau^{2}10b^{1}15^{1}$ / $10b^{1}15^{2}$	582	$\tau^{2}6a^{1}$ / $\tau^{3}10b^{1}15^{1}$ / $\tau^{1}10a^{2}$ / $\tau^{1}10b^{4}$
613	$\tau^{1}10b^{1}6a^{1}$ / $\tau^{2}10a^{1}15^{1}$ / $\tau^{2}10b^{2}15^{1}$ / $10b^{2}15^{2}$	646	$10b^{2}6a^{1}$ / $\tau^{1}6b^{1}$ / $\tau^{3}15^{2}$
677	$\tau^{3}6a^{1}$ / $\tau^{1}15^{1}6a^{1}$ / $\tau^{2}10b^{1}15^{2}$ / $10b^{4}15^{1}$ / ν_{s}^{1}	710	$\tau^{3}10a^{1}15^{1}$ / $\tau^{1}10a^{1}15^{2}$ / $\tau^{1}10a^{2}10b^{1}$ / $\tau^{2}10b^{1}6a^{1}$ / $10b^{1}15^{1}6a^{1}$ / $\tau^{1}10b^{2}15^{2}$
742	$\tau^{1}10a^{1}6a^{1}$ / $\tau^{1}10b^{2}6a^{1}$ / $\tau^{2}6b^{1}$ / $10b^{3}15^{2}$ / $\tau^{1}10a^{1}10b^{1}15^{1}$	804	$6a^{2}$ / $\tau^{3}10b^{1}6a^{1}$ / $\tau^{2}10b^{2}15^{2}$ / $10b^{2}6b^{1}$ / $10b^{1}\nu_{s}^{1}$
(k) via S_1 $\tau^{2}6a^{1}$ (418 cm^{-1})			
400[a]	$6a^{1}$ / $\tau^{1}10b^{1}15^{1}$	495	$\tau^{1}6a^{1}$ / $\tau^{2}10b^{1}15^{1}$ / $10b^{1}15^{2}$ / $16a^{1}$
525	$10b^{1}6a^{1}$ / $\tau^{1}10b^{2}15^{1}$ / $\tau^{1}10a^{1}15^{1}$	581[a]	$\tau^{2}6a^{1}$ / $\tau^{3}10b^{1}15^{1}$ / $\tau^{1}10a^{2}$ / $\tau^{1}10b^{4}$
613[a]	$\tau^{1}10b^{1}6a^{1}$ / $\tau^{2}10a^{1}15^{1}$ / $\tau^{2}10b^{2}15^{1}$ / $10b^{2}15^{2}$	674	$\tau^{3}6a^{1}$ / $\tau^{1}15^{1}6a^{1}$ / $\tau^{4}10b^{1}15^{1}$ / $\tau^{2}10b^{1}15^{2}$ / ν_{s}^{1}
738	$\tau^{1}10a^{1}6a^{1}$ / $\tau^{1}10b^{2}6a^{1}$ /	782	$\tau^{4}6a^{1}$ / $\tau^{2}15^{1}6a^{1}$ / $15^{2}6a^{1}$

	$\tau^2 6b^1$ / $10b^3 15^2$ / $\tau^1 10a^1 10b^1 15^1$		/ $\tau^3 10b^1 15^2$
806	$6a^2$ / $\tau^3 10b^1 6a^1$ / $\tau^2 10b^2 15^2$ / $10b^2 6b^1$ / $10b^1 \nu_s^1$		
	(l) via S_1 ν_s^1 (658 cm^{-1})^b		
684	ν_s^1	715	$7a^1$
767	$\tau^1 \nu_s^1$	856	$10b^1 7a^1$
874	$\tau^2 \nu_s^1$	905	$\tau^1 10b^1 \nu_s^1$
	(l) via S_1 ν_s^1 (666 cm^{-1})^b		
673	ν_s^1	719	$7a^1$
765	$\tau^1 \nu_s^1$	816	$10b^1 \nu_s^1 / \tau^1 7a^1$
849	$10b^1 7a^1$	863	$\tau^2 \nu_s^1$
898	$\tau^1 10b^1 \nu_s^1$		
	(m) via S_1 $\tau^1 \nu_s^1$ (688 cm^{-1})^b		
683	ν_s^1	771	$\tau^1 \nu_s^1$
817	$10b^1 \nu_s^1$	865	$\tau^2 \nu_s^1$
911	$\tau^1 10b^1 \nu_s^1$		
	(o) via S_1 $\tau^2 \nu_s^1$ (734 cm^{-1})^b		
685	ν_s^1	722	$7a^1$
818	$10b^1 \nu_s^1 / \tau^1 7a^1$	855	$10b^1 7a^1$
915	$\tau^1 10b^1 \nu_s^1$	941	$\tau^1 10b^1 7a^1$
	(p) via S_1 $\tau^1 12^1$ (824 cm^{-1})^b		

821	12^1	911	$\tau^1 12^1$
950	$10b^1 12^1$	1001	$\tau^2 12^1$
1047	$\tau^1 10b^1 12^1$		

[a] These values have strong intensities in each SEVI spectrum.

[b] Tentative assignments due to noisy spectra.

5. Global minimum geometries in S_1 state by several *ab initio* calculations

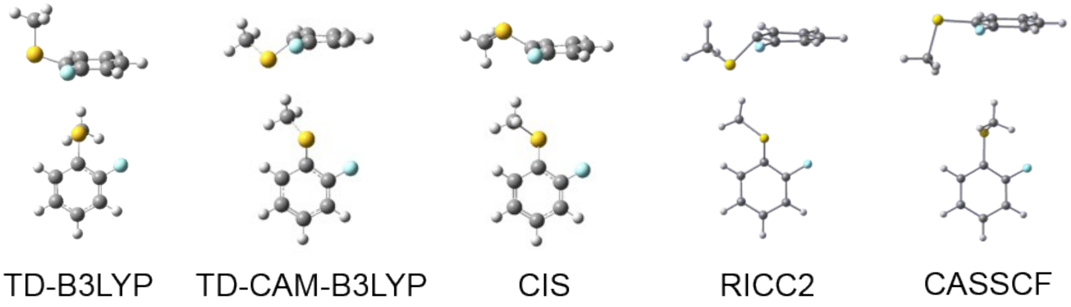

Figure S5. Optimized S_1 geometries obtained using TD-B3LYP/6-311++G(3df,3pd), TD-CAM-B3LYP[6]/6-311++G(3df,3pd), CIS[7]/6-311++G(d,p), RICC2[8, 9]/aug-cc-pVDZ, and CASSCF[10, 11] (10,9)/6-311++G(d,p). All calculations were conducted using Gaussian 09[12], except RICC2 (Turbomole v7.0.2[13, 14]).

6. Velocity-map ion images and deconvolution of total translational energy distributions

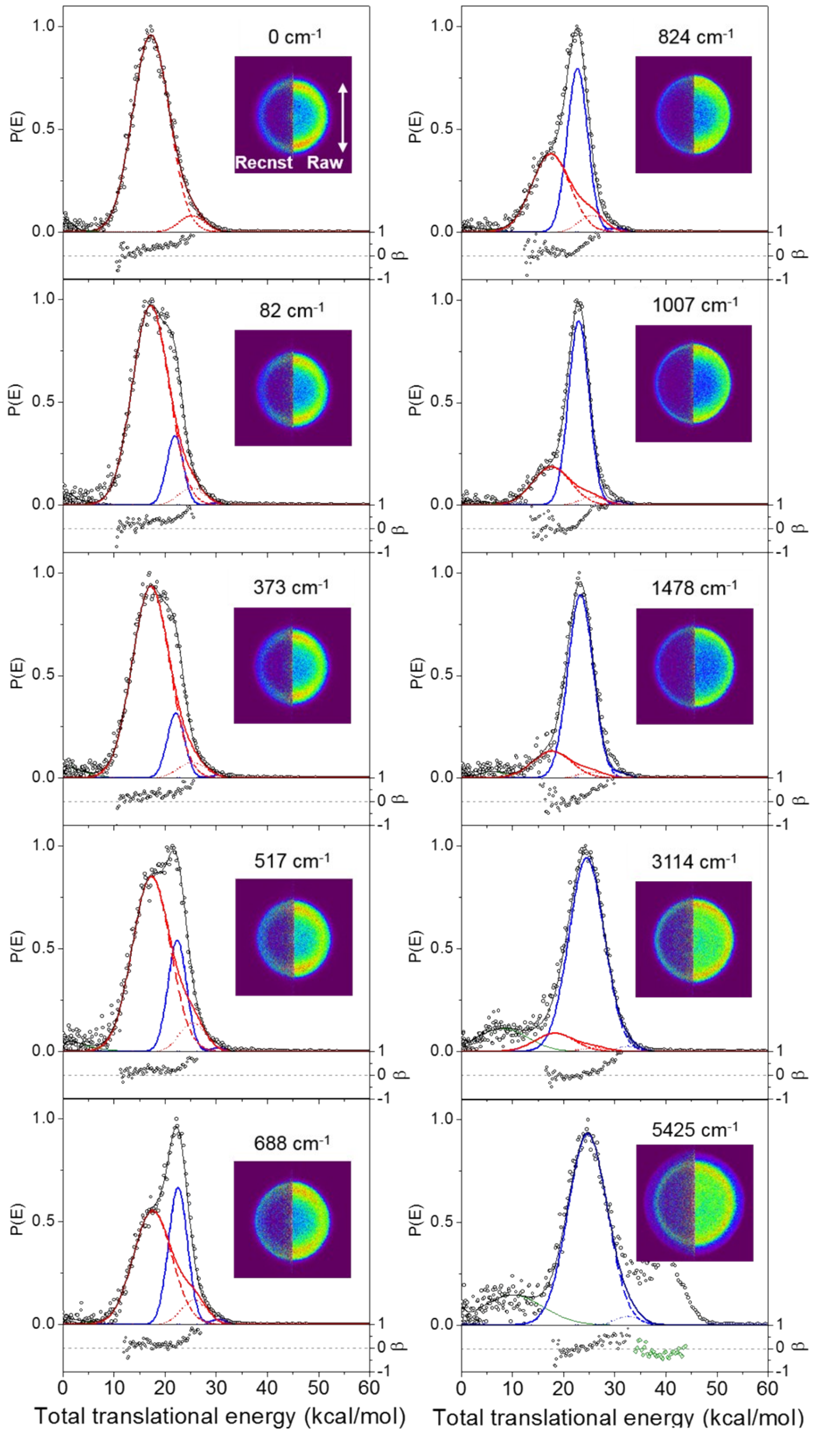

Figure S6. Raw and reconstructed CH_3 images and corresponding total translational energy distributions (black circles) at selected vibronic transitions. The white arrow indicates the polarization direction of the pump pulse. The red (blue) line is the sum of two Gaussian functions of the dashed and dotted lines which correspond to Ã (dashed lines) and $\tilde{X}$ (dotted lines)

states, respectively, of the low (high) translational energy channel. The sum of all the fitted lines including the statistical background (olive lines) is in black line. The anisotropy parameters (βs) shown here are the weighted averaged and the resultant values are plotted in Figure 5(b).

Fitting procedure

The total translational energy distribution was first subtracted by the background for multiphoton dissociative ionization. This background at the lowest translational energy was fitted as a Boltzmann-like function:

$$P_{bg1}(E) = A_1\left(\frac{E}{\pi C_1^3}\right)^{\frac{1}{2}} e^{\left(\frac{-E}{C_1}\right)} \qquad \text{(Equation S1)}$$

where A_1 is the amplitude and C_1 is the width. Above the 1345 cm^{-1} S_1 internal energy, a Gaussian function was further included for the background at the center of the image:

$$P_{bg2}(E) = \left(\frac{A_2}{C_2\sqrt{\pi/2}}\right) e^{\left(\frac{-2(E-B_2)^2}{C_2^2}\right)} \qquad \text{(Equation S2)}$$

where A_2, B_2, and C_2 are the amplitude, center, and width, respectively. The type of the function was determined by a series of imaging experiments at the same excitation wavelength.

It was assumed that the total translational energy distribution at the S_1 origin has only the low translational energy channel (I) so it was fitted as two Gaussian functions (the same form as Equation S2) corresponding to the $\tilde{X}$ and Ã states of 2-fluorothiophenoxyl radical. The $\tilde{X}$-Ã energy gap was fixed at 8.006 kcal/mol (2800 cm^{-1}) in the whole fitting procedure in order to reduce the number of variable. The distribution at the origin was fitted as a Gaussian function centered at 17.2 kcal/mol, covering the majority of the distribution under 24.368 kcal/mol (which is the maximum translational energy of the Ã state). The left (small) distribution was fitted and assigned as the $\tilde{X}$ state. The rest of the distribution at the 32 and 82 cm^{-1} S_1 internal energy after the subtraction of fits at origin (both the $\tilde{X}$ and Ã states) was assigned to the high translational energy channel (II) which also has both the $\tilde{X}$ and Ã states. The position of $\tilde{X}$ state of the low

energy channel overlaps with that of the high energy channel, causing to increase the uncertainty in the $\tilde{X}$/Ã ratio of the low energy channel. We assumed that the $\tilde{X}$/Ã ratio increases with the increase of the excitation energy and the ratio for the low energy channel was restricted to 0.14 referred to the value for thioanisole. The center and the width of all the Gaussian functions increase as the excitation energy increases.

The error range of the fraction (Γ) of the high translational energy channel in the total distribution was obtained by the repetition of the imaging experiment and a fitting procedure by adjusting the relative amount of the Ã states of the low and high translational energy channel. The final error bar was determined as the largest one.

7. UV absorption spectra in hexane

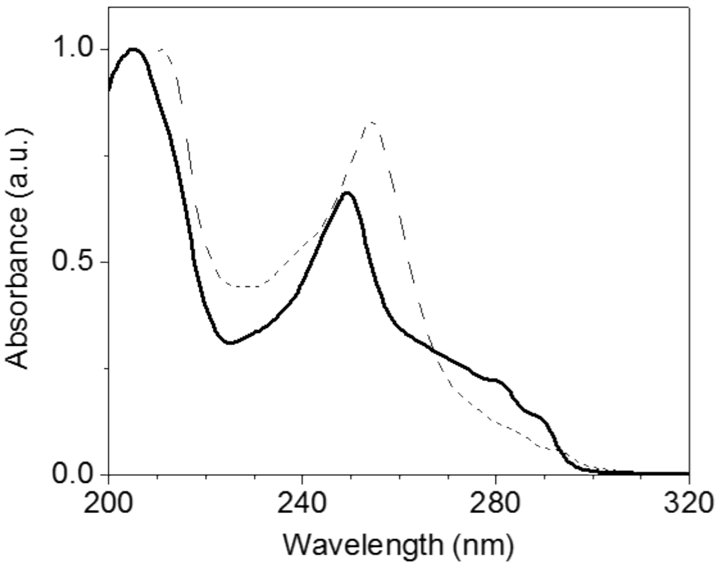

Figure S7. UV absorption spectrum of 2-fluorothioanisole (solid line) compared to that of thioanisole (dashed line) in n-hexane at room temperature.

REFERENCES

1. B. Schmidt and U. Lorentz, *WavePacket 4.7:A Program Package for Quantum-Mechanical Wavepacket Propagation and Time-Dependent Spectroscopy*, available via http://wavepacket.sourceforge.net.
2. A. D. Becke, *Physical Review A*, 1988, **38**, 3098-3100.
3. All Franck-Condon simulations have been carried out using FC-LabII Version 2009.a, a computational package developed by C. Schriever, M.C.R. Cockett and I. Pugliesi. The lastest information on program updates, a basic introduction to Franck-Condon simulations and a free download of the software can be found at http://www.fclab2002.net/.
4. I. Pugliesi and K. Müller-Dethlefs, *The Journal of Physical Chemistry A*, 2006, **110**, 4657-4667.
5. G. Varsányi, *Assignments for Vibrational Spectra of 700 Benzene Derivatives*, Wiley, New York, 1974.
6. T. Yanai, D. P. Tew and N. C. Handy, *Chemical Physics Letters*, 2004, **393**, 51-57.
7. J. B. Foresman, M. Head-Gordon, J. A. Pople and M. J. Frisch, *The Journal of Physical Chemistry*, 1992, **96**, 135-149.
8. O. Christiansen, H. Koch and P. Jørgensen, *Chemical Physics Letters*, 1995, **243**, 409-418.
9. F. Weigend, M. Häser, H. Patzelt and R. Ahlrichs, *Chemical Physics Letters*, 1998, **294**, 143-152.
10. P. J. Knowles and H.-J. Werner, *Chemical Physics Letters*, 1985, **115**, 259-267.
11. H. J. Werner and P. J. Knowles, *The Journal of Chemical Physics*, 1985, **82**, 5053-5063.
12. M. J. Frisch, G. W. Trucks, H. B. Schlegel, G. E. Scuseria, M. A. Robb, J. R. Cheeseman, G. Scalmani, V. Barone, B. Mennucci, G. A. Petersson, H. Nakatsuji, M. Caricato, X. Li, H. P. Hratchian, A. F. Izmaylov, J. Bloino, G. Zheng, J. L. Sonnenberg, M. Hada, M. Ehara, K. Toyota, R. Fukuda, J. Hasegawa, M. Ishida, T. Nakajima, Y. Honda, O. Kitao, H. Nakai, T. Vreven, J. A. Montgomery, J. E. Peralta, F. Ogliaro, M. Bearpark, J. J. Heyd, E. Brothers, K. N. Kudin, V. N. Staroverov, R. Kobayashi, J. Normand, K. Raghavachari, A. Rendell, J. C. Burant, S. S. Iyengar, J. Tomasi, M. Cossi, N. Rega, J. M. Millam, M. Klene, J. E. Knox, J. B. Cross, V. Bakken, C. Adamo, J. Jaramillo, R. Gomperts, R. E. Stratmann, O. Yazyev, A. J. Austin, R. Cammi, C. Pomelli, J. W. Ochterski, R. L. Martin, K. Morokuma, V. G. Zakrzewski, G. A. Voth, P. Salvador, J. J. Dannenberg, S. Dapprich, A. D. Daniels, Farkas, J. B. Foresman, J. V. Ortiz, J. Cioslowski and D. J. Fox, Gaussian 09 (Revision D.01), Gaussian Inc., Wallingford, CT, 2009.
13. TURBOMOLE V7.0.2 2015, a development of University of Karlsruhe and Forschungszentrum Karlsruhe GmbH, 1989-2007, TURBOMOLE GmbH, since 2007; available from http://www.turbomole.com.
14. R. Ahlrichs, M. Bär, M. Häser, H. Horn and C. Kölmel, *Chemical Physics Letters*, 1989, **162**, 165-169.
